# Supplementary material for: Paternally expressed imprinted genes establish postzygotic hybridization barriers in Arabidopsis thaliana
Source: eLife. 2015 Sep 7;4:e10074. doi: 10.7554/eLife.10074 (PMC4589659; doi:10.7554/eLife.10074)
Supplement: Supplementary file 1. — Primers used in this study. DOI: http://dx.doi.org/10.7554/eLife.10074.018 [file elife10074s002.pdf]

Supplementary Table 1: Primers used in this study

Gene	Genotyping	Expression	Imprinting
PEG1	TTTACCCGTCTTGTCGATTTG<br>TTCCAGTGGTTTTGGTAGACG	ATCTCCAATAACCACTACAG<br>TCATAACCACCAGAAACAGAA	
PEG2	TGGTGATGATTTATGGATTTTAAC<br>ACAAGGTACGCGATTATGTCG	AGGACCAACATTATGGTCATACC<br>GGAAGTGATAGAAGCGGTAGAG	AGGGAGAAACAATTGGCTTTAGA<br>CTATCACTTCCCCGAATCTCCTA
	PEG2::PEG2 cloning:<br>CACCTCAACAACCTAAGAGATCACACCA<br>AAAATGAGTAGAGGATGAAGCTGGAT		
PEG3	TCGAATGAAGAGGACAAACAAC<br>TAAACAAACCGATTCTGACGG	CGAACGTTAGAGGTCATCCG<br>GCAACAACCAAGAAAGACTGG	ATCAGAAATTTTCTCACCGGGA<br>TGAAACTGGTGTCTTCTGCTGAAC
PEG4	TCTCCATGGAGAATGTTGGAG<br>AAGGAATCGGCTTGTAATTCG	CATGGAGAATGTTGGAGGAACG<br>ATTGTCACACTGATAGATAGGAGC	ACACTGTTGGAGCTTGATCTTCTG<br>TGGTTCAGGGCAGTAGCAAAG
PEG5	AACCATTTACAACGGTCACTAGC<br>TCAAACCCATCTGAATTCTCG	GATATCTGAGAGCGGAGGAG<br>AATCACAACTTGGACTGGAC	
PEG6	TGATTGCTTGGTTATTCTCCC<br>CTGTCACGGAAATCCCTAGTG	GAGCCTGGTCATCAACTAAACC<br>TAGAAGATCAGCACAGGAACG	AGGATTCTCCCTACAACCTTCTACA<br>GATTATATGCATCATCCTCGTCG
PEG7	ACGGTAATGGCCCAAATTTAG<br>CTTCGCACTGATTTTCTGGTC	TTTGTGAACCAGGGCAAGAG<br>ATTCGAGACGAGTCTTTGAGTC	GCCTATCGAATATTCCTGAT<br>GCTTGATCTTCTTAGGTTTC
PEG8	AGAACCAAAACCGAACCAAAC<br>TCGGGTCTTGTTTTGTTTCAC	TTACCGATAGGAATCTGAGAAGTC<br>CATAGGCGGCCTAAGGAGTTTAG	
PEG9	GAAAGTCTATGTCCACGAGCG<br>GAAGAAATTCAAATCCTCGGC (*peg9-1*)	GCTCAAGAAGGGAAATCTAACTG<br>AAACCTTGTCGGTCTTGTAGG	
	GAAAGTCTATGTCCACGAGCG<br>ACGAGCTCAAGAAGGGAAATC (*peg9-2*)		
ADM	ATTGACATTTCCTTGGGGTTC<br>ATATTTCTCGTTGGGGACCAG (*adm-2*)	TTGAAAGAGTTTGCGGATGTG<br>AGGACCAACATTATGGTCATACC	
FXG1	TTCCCCATAGCTAAAAGAGGC<br>CTCCTCCACGACTACGAACAG	CTTCATAGCGGAGAGTCTTGG<br>TTGACGTAGAGTAGAGTTAAGAGC	
HDG10	AAGCGTGTAATCACAAAGTTTG<br>TGTCCCCAAAAAGTTGAAAATC	GGTCGTTCTAAGGTAACATGGA<br>TCCTCTGAAGAGTAGCGGTC	GGTCGTTCTAAGGTAACATGGA<br>TCCTCTGAAGAGTAGCGGTC
PKR2	TTCTGATTTTTCAACCGATGG<br>GGGGAGGAGTATCTGGTGAAG	ATGCTGATAGAACAAGTCACTGG<br>CATACATCACCTGCTTGCCG	
SUVH7	CTCTTGATCACGAGCGTTACC<br>TGCCATGTTTCGTAAATCTCC (*suvh7-1*)	TGCTGGTCCTAGTAGTACTGGTCCA<br>CACCCGCATCACAAGAATAGTTG	GTGAAATGTGCTTAGTGGGGCTT<br>CCTCTCACGACTCTAACGTCATTCC
	CAGTTTCTGTCTCTGCTTCGG<br>CCGCAAAATTCTCCATTTCTC (*suvh7-2*)		
	TTATTGGTTTGTGATTTCGTCG<br>AACCATTGATTTACGAGTGCG (*suvh7-3*)		
VIM5	TATATGGAAGAAGGTAATGAAATA<br>TCAAAAGAAATCATGGCAACC	GTTGTCAGGTCTACCAAGGA<br>AAACGGCACATCTGTATTCCA	
YUC10	CCTGAATCTCGCCATCGCGAATC<br>CCAAAGAGCTTTTCGTCAACTACC	GCGAGATTCAGGTTATCAATGG<br>GTAACCAGTAGCAAACACAATCG	

Actin11		AACTTTCAACACTCCTGCCATG<br>CTGCAAGGTCCAAACGCAGA	
AGL35		GGAACAATGAACCATCTTCC<br>AAGAAGAACCCATCTCAACC	
AGL36		GTGCTCTCATCTACAGTCCA<br>CATCATCTTCTTGGTTCGGG	
PHE1		TCCAACACCGAAAACTCCAT<br>CGCATGTGCGGTCATCC	
AGL40		AATATACTCACTGAGGTGCTG<br>ACCAGTTTCCTACTTGTTCTC	
AGL62		CCTCCTCACCAACACAACAA<br>ACCTTTGAACCCCTCGAGTT	
AGL90		CTTGTGCTCTCATCTATAGTCCA<br>GTTTCTTGATCCATCATCTTCCTG	
AGL96		GTGAACTTGCTGATCTCTCCCC<br>GCTCTCTCCTTTATAGGTCCCCAA	
